# Anthropogenic effects on a tropical forest according to the distance from human settlements

**DOI:** 10.1038/srep14689

**Published:** 2015-10-05

**Authors:** Ananya Popradit, Thares Srisatit, Somboon Kiratiprayoon, Jin Yoshimura, Atsushi Ishida, Masae Shiyomi, Takehiko Murayama, Pranom Chantaranothai, Somkid Outtaranakorn, Issara Phromma

**Affiliations:** 1 Inter-Department of Environmental Science, Graduate School of Chulalongkorn University, Phayathai Rd. Phatumwan, Bangkok 10330, Thailand; 2 Department of Environmental Engineering, Faculty of Engineering, Chulalongkorn University, Phayathai Rd, Phatumwan, Bangkok 10330 Thailand; 3 Center for Ecological Research, Kyoto University, Otsu, Shiga 520-2113, Japan; 4 Department of Mathematical and Systems Engineering and Graduate School of Science and Technology, Shizuoka University, 3-5-1 Johoku, Naka-ku, Hamamatsu 432-8561, Japan; 5 Marine Biosystems Research Center, Chiba University, Kamogawa 299-5502, Japan; 6 Department of Environmental and Forest Biology, State University of New York College of Environmental Science and Forestry, Syracuse, NY13210, USA; 7 Department of Environmental Science, Faculty of Science and Technology, Thammasat University, 99 Moo 18 Paholyothin Road, KlongLuang, Rangsit, Prathumthani 12121, Thailand; 8 Faculty of Science, Ibaraki University, Mito, Ibaraki 310-8512, Japan; 9 Department of Environmental Science and Technology, Interdisciplinary Graduate School of Science and Engineering, Tokyo Institute of Technology, Nagatsuta, Midori-ku, Yokohama City, Kanagawa, 226-8502, Japan; 10 Department of Biology, Faculty of Science, KhonKaen University, 123 Moo 16, Mittraphap Road, Muang, KhonKaen 40002, Thailand; 11 Department of National Parks, Wildlife and Plant Conservation, 61 Pholyothin Road, Ladyao, Chatuchak, Bangkok 10900, Thailand; 12 Department of Environmental Science, Faculty of Science, KhonKaen University, 123 Moo 16, Mittraphap Road, Muang, KhonKaen 40002, Thailand

## Abstract

The protection of tropical forests is one of the most urgent issues in conservation biology because of the rapid deforestation that has occurred over the last 50 years. Even in protected forests, the anthropogenic effects from newly expanding villages such as harvesting of medicinal plants, pasturing cattle and forest fires can induce environmental modifications, especially on the forest floor. We evaluated the anthropogenic effects of the daily activities of neighboring residents on natural forests in 12 plots extending from the village boundary into a natural forest in Thailand. The basal area per unit land area did not present a significant trend; however, the species diversity of woody plants decreased linearly towards the village boundary, which caused a loss of individual density because of severe declines in small saplings compared with adult trees and large saplings in proximity to the village. An analysis of tree-size categories indicates a lack of small samplings near the village boundary. The current forest appears to be well protected based on the adult tree canopy, but regeneration of the present-day forests is unlikely because of the loss of seedlings.

Anthropogenic impacts and global climate change are one of the most critical issues on the conservation of forest ecosystems not only in developing countries^1^ but also in developed countries^2^. The rapid population growth in developing countries has increased the demands in forest exploitation in the tropics^1^. Many people have migrated from urban areas to establish new villages in tropical forests, because of rapid population increase in cities for the last several decades^3^,^4^. Tropical forests have been heavily exploited by those immigrants. Urban immigrants, unlike indigenous people, have no traditional knowledge or skill on the sustainable use of natural forests, causing severe damage to forest ecosystems^5^,^6^. The rapid expansion of these existing communities into protected areas is expected to cause severe anthropogenic degeneration and fragmentation of natural forests, further deteriorating the forest structure and biodiversity of important natural tropical forests^4^,^7^,^8^. Such anthropogenic impacts result in the degradation of ecological services received from forest ecosystems.

Protected areas in Thailand were first established approximately 50 years ago. Since then, the Thai government has tried to protect natural tropical forests, e.g., construction of network corridors^9^. Yet, the Thai government is not fully successful on the conservation efforts^10^,^11^,^12^. The present study site has been designated a protected area for the last 20 years (Fig. 1). There are three villages in the mixed deciduous forests (MDF) area in the Phu Kao–Phu Phan Kham National Park in Thailand. Despite its protected status, village areas have been expanded more rapidly in the last 20 years than in previous years (Fig. 1). These natural forests have high economic value from medicinal trees and as food banks. Illegal logging is strictly prohibited in the protected areas of Thailand. However, partial cutting of leaves, bark and roots of medicinal plants, and harvesting of edible fruits are still being practiced. Many studies to assay forest degradations use satellite images, because of the ease of broad surveys^13^. However, in such protected forests, anthropogenic effects are not easily detected from satellite images because of their dense canopy. Although these studies have not detected any anthropogenic impacts, continuous exploitation of medicinal and commercially valuable plants can severely affect forest ecosystems.

In Southeast Asia, MDF is a major tropical dry forest^14^,^15^,^16^. It experiences a distinct dry season for several months each year. Frequent forest fires happen during this dry months, killing small tree saplings. Harvesting medicinal plants and edible fruits, and pasturing cattle are also frequently conducted within the protected forests. To evaluate these anthropogenic impacts on the MDF in the national park, we analyzed the forest composition, species diversity, and natural regeneration in MDF according to the distance (200 to 2000 m) from the village boundaries.

To understand the present condition of the study forest, dominance (basal area per ha), the number of species per unit area, and some indices related to biodiversity were examined along the distance from the village boundary to the natural forests. To estimate forest regeneration and resulting future forests, the continuity from sapling to adult tree in each species was also examined. The conditions of medicinal woody plants and pioneer trees were separately analyzed, because they were suspected to be the first sign of forest damage in the current study forest. According to these variables, we can demonstrate how forest degradation worsens as the distance gets closer to the villages. Furthermore, based on species composition of adult and juvenile trees, we found out that the woody species have a high risk of disappearing in the future.

## Results

### Species structure and individual distribution of woody plants

The study site exhibits a high biodiversity of woody plants (see the Methods section on the survey methods and measurements). The total number of individual woody plants was 4211 in the entire research area (3 ha), and there were 148 species within 65 families (Table S1). Out of 148 tree species, 88 were medicinal species and 9 were pioneer species (Table S2). The mean density was 1,416 individuals ha^−1^, and the mean total basal area was 20 m^2^ ha^−1^. We calculated the important values (IVs) in each species in each plot (2500 m^2^ in land area). IV in a species is the sum of the relative frequency (*F*: percentage of the number of species to the total species), the relative individual density (*D*: percentage of the number of individuals in each species to the total number of individuals), and the relative basal area (*BA*: percentage of the basal area covered by a species to the total basal area) (see Equation (1) in Methods)^17^. Throughout the plots, 23 woody plant species were observed with high important values (IV > 11) and recognized as predominant tree species (Fig. S1 and Table S3). Based on the variation of Morisita’s dispersion index (*I*_*δ*_), all of the predominant tree species exhibited a clumped distribution (*I*_*δ*_ > 1; Table S4), meaning that the distance between neighboring individuals was minimized for each species^18^. No obvious trend was observed in the spatial pattern of the predominant tree species with high IV values along with distance from the boundary lines (Figs S1 and S2).

The total basal area per a unit land area is an indicator of tree dominance in survey plots because it correlates with the maturity of forests. In this survey, a significant trend in total basal area per ha (Dominance) with distance from the village boundaries to the natural forests was not observed (Fig. 2a). However, the tree species composition was significantly different depending on the distance from the village boundaries. The numbers of total woody plant species and medicinal tree species significantly (*P* < 0.001) increased linearly with the distance from the village boundaries toward the forest (Fig. 2b). However, no significant trend was found between the number of pioneer tree species and the distance; the average number of pioneer tree species per 2500 m^2^ in land area was 5.4 (Fig. S3).

To evaluate the contribution of these functional types (medicinal and pioneer trees) on the community structure of woody vegetation, their IV and its determinants (*F*, *D* and *BA*) in each plot were calculated (Fig. 3). The IV of medicinal trees increased significantly (P < 0.01) and that of pioneer trees decreased significantly (P < 0.05) with the distance from the village boundaries toward the forest. In the medicinal trees, an increase in all *F*, *D* and *BA* (i.e. relative number of species, relative number of individuals and relative basal area, respectively) contributed to the increase in IV. In the pioneer trees, a decrease in only *F* (i.e. relative number of species) contributed to the decrease in IV.

### Species diversity and anthropogenic impact

To examine the variations of biodiversity in woody plants among the plots, the Shannon–Wiener Index (*H′*), Fisher’s index (*α*), and Simpson’s index (*λ*) were calculated for each plot (Fig. 4 and Supplementary Table 4). The values of *H′* increased significantly with distance from the boundary lines (*P* < 0.01 in overall; Fig. 4a). Here, all of the examined plots showed high *H*′ values (>3.87) (see Table S5), indicating that high biodiversity still remains in the study area compared with the usual range of *H*′ values in natural forests (between 1.5 and 3.5 and rarely surpassing 4.5)^19^,^20^. Fisher’s *α* also increased significantly with distance from the boundary lines (*P* < 0.01 in overall; Fig. 4b). As expected, *H*′ was positively (*p* = 0.0001) correlated to Fisher’s *α*. Simpson’s index (*λ*) is another measurement of biodiversity ranging between 0 (highest diversity) and 1 (no diversity). The values of (1 − *λ*) were similar or all plots (closed to 0); thus biodiversity was observed in all plots, and a trend along with distance from the villages was not observed (Fig. 4c). Significant differences were not observed in the values of (1 − *λ*) among all twelve plots (analysis of variance (ANOVA), *P* = 0.69). Note that the southern direction exhibits anomalies in all three biodiversity indices, and the intermediate plots exhibit a peak value.

To evaluate the distribution patterns of individual trees, we examined the evenness index (*E*), which decreased significantly with distance from the boundary lines (*P* < 0.001 in overall), indicating that the individual trees of all species tend to be regularly distributed in the plots close to the villages compared with remote plots.

### Forest regeneration

Sapling trees on the forest floor are an important determinant of forest regeneration. In each plot, we surveyed individual trees and categorized them into three size classes: (1) adult trees (diameter of breast height (DBH) ≥ 45 mm), (2) large saplings (20 mm ≤ DBH < 45 mm), and (3) small saplings (10 mm ≤ DBH < 20 mm). For the adult trees and large saplings, the number of species, density of individual trees, variance of frequency, and log total basal area per unit land area were not correlated with distance from the villages (Fig. 5). This indicates that the prohibition law against tree logging has been enforced well in the protected area for the last 20 years. However, for the small saplings, these values increased significantly with distance (*P* < 0.001 for number of species, density of individual trees, and variance of frequency; and *P* < 0.01 for log total basal area). In close proximity to the villages, the individual density of small saplings was very low (0.24 m^−2^ and 1.2 m^−2^ in Plot N1 and E1, respectively; see Fig. S2 and Table S5 for plot locations), indicating a scant forest understory. In addition, the percentage of small saplings to the number of total individual trees was 6.7% (Plot N1) and 17.3% (Plot E1), indicating a shortage of juvenile trees in these plots.

The successful regeneration of a tree species should depend on whether there are enough number of individuals found in each size class from small saplings to adult trees. To estimate the future progression of forest regeneration, the number of tree species in each size class and overlapped size-classes for each plot are shown using an Euler diagram (Fig. 6). In the diagram, the overlap in numbers of species of different sizes provides a good estimate of regeneration success. If the number of overlapping species among all size classes is high, natural regeneration is expected to be successful (Plots N3, E3, W2, W3, S2 and S3, which are remote from the villages). However, if the number of overlapping species among all size classes is low, then future regeneration of adult trees might be disturbed, and result in the drastic changes to the composition of canopy-tree species (Plots N1 and E1, which are close to villages). Furthermore, if the numbers of overlapping species between large and small saplings are also low, forest regeneration might fail over time (Plots N1 and E1).

In some tree species, individuals were found in all size classes indicating successful regeneration in future. For example, in all directions, *Xylia xylocarpa*, *Bauhinia saccocalyx, Pterocarpus marcocarpus,* and *Cananga odorata* had enough individual trees in all size classes in all plots. In contrast, some tree species were found only in the adult-tree class, indicating that these species are likely to disappear in future, e.g., *Calycopteris floribunda* (IV = 0.82) in Plot N1, *Sauropus androgynus* (IV = 0.46) in Plot N2, *Dialium cochinchinense* (IV = 0.4) and *Excoecaria oppositifolia* (IV = 0.51) in Plot N3, and *Dalbergia* sp. (IV = 0.82) in Plot S1, which are all non-predominant species. Trees species that were only observed in the large sapling class in a plot might also be under the risk of extinction, including *Anthocephalus chinensis* (IV = 0.27) in Plot N3, *Gmelina philippensis* (IV = 0.19) in Plot W2, and *Dalbergia cochinchinensis* in Plot N3, W1, and W2. All these species except for *D. cochinchinense* are medicinal woody plants.

## Discussion

The current study shows conflicting results. First, there are little or no apparent anthropogenic effects in terms of total basal area, Simpson’s index (λ), and numbers and density of adult trees and large saplings (Figs 2a, 3c and 4). This finding may suggest that the study forest is fairly well maintained irrespective of distance from the villages. However, with respect to the future regeneration of the forest, a drastic effect was observed on the recruitment of trees in the studied forest (Figs 5 and 6). The species compositions within size classes exhibited a failure to regenerations for a number of species in close proximity to the villages (Fig. 6). These tree species are likely to be degraded by anthropogenic effects in the future because of a lack of saplings in close proximity to the villages.

Four major human-induced factors with significant effects on the biodiversity of tropical have been proposed: 1) deforestation and fragmentation, 2) over-exploitation, 3) invasive species, and 4) climate change^21^,^22^. Excessive land use by local residents will promote deforestation and forest fragmentation, including edge effects of forests^23^. Harvesting medicinal plants and edible fruits, trampling forest soil, and grazing by domestic animals (mostly cattle in Thailand) can reduce the seedling stock on the forest floor^24^. Forest fires usually occur during the dry season, killing tree saplings. All of these human activities may prevent seedling recruitment by altering the microhabitat of the forest floor and soil conditions, e.g., increasing dry conditions and destroying soil fauna, including mycorrhizal fungi. Furthermore, environmental changes at the forest floor, such as increasing solar exposure and decreasing CO_2_ concentrations will cause a reduction in carbon gain and result in degradation of the biodiversity of understory plants and tree seedlings^25^. Although illegal logging of large trees are well prohibited, such anthropogenic disturbances to the forest floor and soil conditions are still critical factors that affect seedling recruitment and subsequent forest regeneration. Here IV and their determinants (*F*, *D* and *BA*) of medicinal trees decreased (Fig. 3a), and IV and *F* of pioneer trees increased (Fig. 3b) near the villages.

Our results indicate that adequate forest regeneration is relatively difficult in close proximity to villages. In addition, human access will be an important determinant of anthropogenic effects. Such conditions may be reflected in plots W1 and W2, where the access is limited by high elevation and steep slopes, thus, allowing the maintenance of a high numbers of species of all tree-size categories of individual trees (Fig. 6). Forest regeneration may be also affected by the interference in seedling recruitment via indirect effects of proximity to human residence. For example, wildlife may avoid human villages and their immediate proximity. As a result, seed dispersal via these animals may become extremely difficult. Such avoidance is observed in elephants^26^,^27^ and a number of other many animal species^28^,^29^.

The present survey was highly limited, investigated relatively small plot size, and only included twelve plots. Therefore, we might not have detected distance effects on dominance (total basal area per 1 ha) (Fig. 2a). By increasing the plot size, for example, we may observe a significant correlation with distance because a slight increase in dominance was found near the villages. The distance from the village boundaries was not evenly placed. Because of this imbalance in plot locations, the detectability of distance effects was greatly reduced. In the mountain this unevenness in plot locations is unavoidable because of geographical constraints and heterogeneity of the target natural forests (MDF) in the study area. Even with these severe limitations, effects are still observed on small saplings and tree species diversity, which suggests that the factors related to small saplings are important for the regeneration and sustainability of tropical forests. The present study illustrates the importance of evaluating the effects of human activities on natural forests in protected areas, where the forest cover does not exhibit any anthropogenic damages.

## Methods

### Study site and plot setting

**Figure 1:**
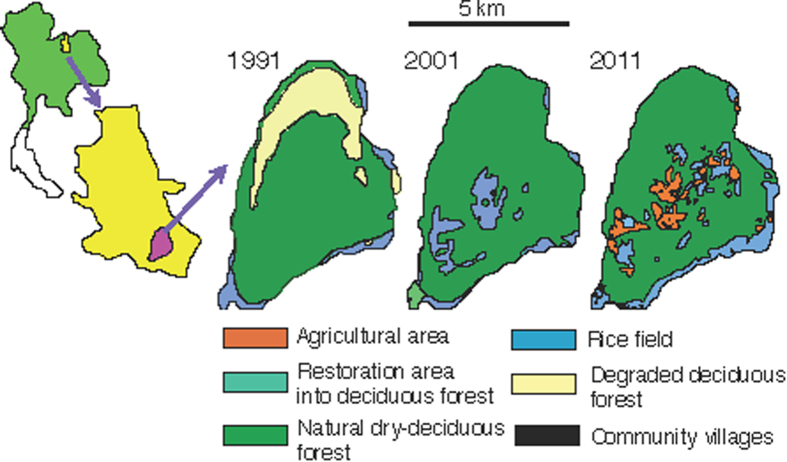
Temporal changes in land use in the Phu Koa (PK) area of Phu Kao–Phu Phan Kham National Park in Thailand. The changes in land use in 1991, 2001 and 2011 (from right to left) over the last 20 years in the protected area included in the study site. The area of degraded forest in 1991 had almost recovered to forests with high vegetation cover by 2001. From 2001 to 2011, the areas of rice fields decreased, but the village and agricultural areas began to expand. We make the figures based on the digital maps of land use in the Land Development Department in Thailand.

**Figure 2:**
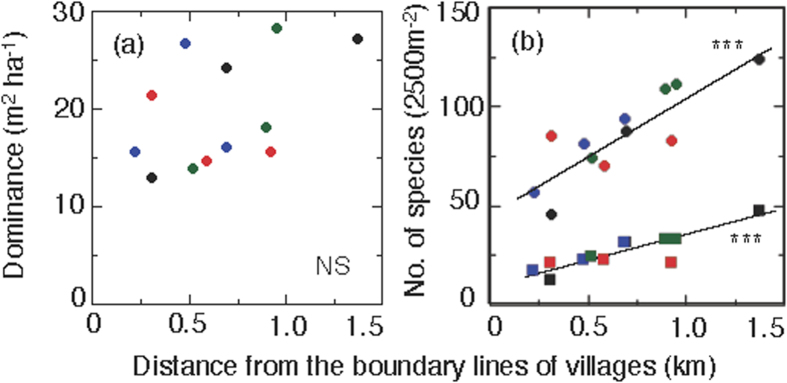
Relationship of dominance and the number of species according to the distance from village boundaries. (**a**) Dominance (=total basal area 1 ha^−1^). (**b**) Numbers of species of all woody plants (circles) and medicinal plants (squares) plotted against the distance from the boundary lines of villages. The direction is indicated in plot colors for east (blue), north (black), south (red), and west (green). Statistical analyses showed that (**a**) is not significant (*r* = 0.479, NS), whereas (**b**) is significant for all woody plants (*r* = 0.855, *P* < 0.001) and for all medicinal plants (*r* = 0.868, *P* < 0.001). See Supplementary Fig. 2 and Supplementary Table 5 for more details on the plot positions.

The current vegetation survey was conduced in an MDF in the Phu Koa (PK) area of Phu Kao–Phu Phan Kham National Park, and the study site covered 318.4 km^2^ and lies between 16 °44′–17 °2′N and 102 °25′–102 °43′E on the Khorat plateau, Thailand (Fig. 1). The average monthly precipitation during the dry season (November to April) was 40.5 mm from 1982 to 2013. The PK area is located on the upper northeastern plateau of the park, and the vegetation consists mainly of MDF, but dry dipterocarp forests and dry evergreen forests partially exist. MDFs have a high biodiversity of woody plants adapted to seasonal drought^14^,^15^, and almost all of the trees are dry-deciduous, which have a period of leaf falling during the dry season^30^,^31^,^32^,^33^. Although the area of the park once was 19.7 km^2^ (5.9% in forest area), only 3.1 km^2^ (0.9% in forest area) of intact MDF remained in 2012^34^.

The PK is shaped like a frying pan with a large plain in its center (Fig. S2). This area is suspected to overlie an extinct volcano that was active several million years ago. The Royal Forestry Department (RFD) and Department of National Park, Wildlife and Plant Conservation (DNP) in Thailand have set the boundary lines of the protected area within the forests. Here, we investigated the effects of village communities and activities of inhabitants on the species composition, species diversity and natural regeneration of woody plants in protected MDF forests.

Three villages are located in the center of the PK. Using a Global Positioning System (GPS; Garmin 60Csx, Garmin, Olathe, KS, USA) and a topographic map, we established the study plots in all four cardinal directions (almost north, west, south, and east) from the village boundary lines into the forests (Fig. S3). In each direction in the MDF, we established three plots (50 m × 50 m, called as L-quadrat) along a transect line, as follows: plots adjacent to village boundaries (Plot 1), plots far from village boundaries (Plot 3), and plots intermediate between the plots (Plot 2). In total, twelve plots were established (three plots in each direction). The range of elevation among the set plots was 235 m to 364 m a.s.l. The straight-line distance from the boundary lines in each plot was derived with ArcGIS 10.2.2 (Environmental Systems Research Institute Inc., Redlands, CA, USA). Detailed information on the position of each plot is shown in Supplementary Fig. 2 and Supplementary Table 5.

### Data collection

Data collection was conducted from November 2013 to February 2014. We established 12 plots with a 50 m × 50 m quadrat (see Supplementary Fig. 2), with a total area of 3 ha. The survey area (3 ha) is sufficient to produce the species-area curve of this MDF^17^. Each large quadrat (50 m × 50 m, L-quadrat) was further divided into 25 sub-quadrats (S-quadrat) of 10 m × 10 m. All woody plants of 10 mm DBH (1.3 m above ground) in each plot were tagged and mapped to the nearest 10 m grid following a standard protocol^35^. In the MDF, frequent wildfires kill many seedlings and saplings that are less than 10 mm DBH during the dry season^36^,^37^,^38^. Therefore, we assumed that only trees and saplings with ≥10 mm DBH can survive a forest fire. We divided the examined individuals into three size categories: (1) adult trees (DBH ≥ 45 mm), (2) large-size saplings (20 mm ≤ DBH < 45 mm) and (3) small-size saplings (10 mm ≤ DBH < 20 mm).

### Data analyses in forest structure and biodiversity

**Figure 3:**
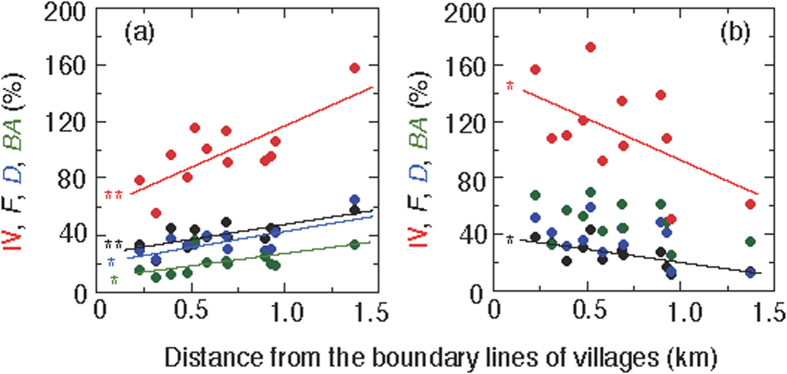
Relationship important value (IV) and its determinants (*F*: relative frequency, *D*: relative individual density, *BA*: relative basal area) to the distance from village boundaries. (**a**) medicinal tree species, (**b**) pioneer tree species. IV: red circles, *F*: black circles, *D*: blue circles, *BA*: green circles. See equation (1) for calculation and parameters (**P < 0.01, *P < 0.05).

**Figure 4:**
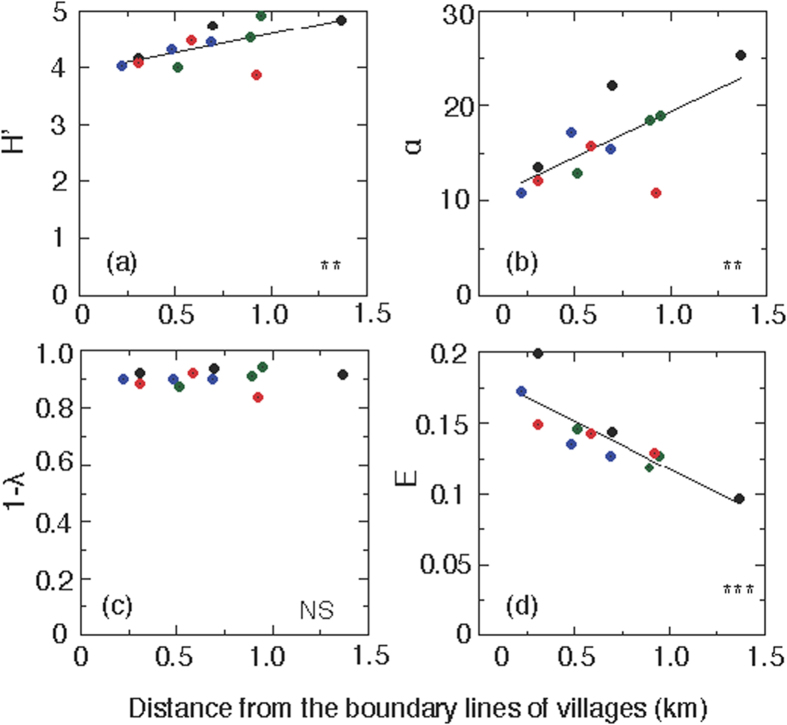
Three biodiversity indices and an evenness index plotted against the distance from village boundaries. (**a**) Shannon–Wiener *H*′ (Σ P_i_ • ln P_i_), (**b**) Fisher’s α (S/ln[1 + N/α]), (**c**) 1-Simpson’s *λ* (1 − ΣP_i_^2^), and (**d**) Hill’s evenness ((1/λ)/*e*^*H′*^) plotted against the distance from the boundary lines of the village. *H*′, 1 − λ, and α describe the overall species diversity, and *E* indicates the distribution pattern of all individual trees in each plot to the east (blue circles), north (black circles), south (red circles), and west (green circles) directions from the village into the forests. Statistical results are as follows: (**a**) (*r* = 0.627, *P* < 0.01), (**b**) (*r* = 0.702, *P* < 0.01), (**c**) (*r* = 0.127, not significant), and (**d**) (*r* = 0.851, *P* < 0.001).

**Figure 5:**
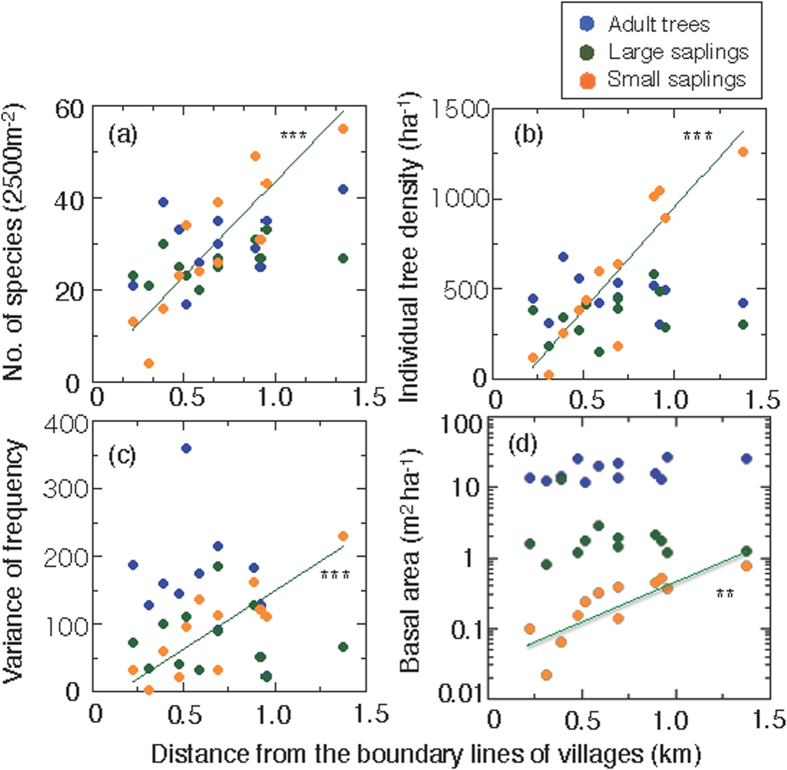
Number of tree species and other characteristics according to distance from village boundaries for three tree-size classes: adult trees, large saplings and small saplings. (**a**) Number of woody plant species, (**b**) density of individual woody plants, (**c**) variance of frequency, and (**d**) total basal area per unit land area, plotted against the distance from the boundary lines of village for adult trees (DBH ≥ 45 mm, blue circles), large saplings (DBH 20 mm ≤ DBH < 45 mm, green circles), and small saplings (DBH < 20 mm, orange circles). For all variables, significant correlations were only found for small saplings: ((**a**) *r* = 0.886, *P* < 0.001), ((**b**) *r* = 0.915, *P* < 0.001), ((**c**) *r* = 0.845, *P* < 0.001), and ((**d**) *r* = 0.821, *P* < 0.01). The variance of frequency indicates the distribution pattern of tree species within a plot; high values indicate that the distribution of all species in the plot differ from each other, whereas low values indicate that all species have a similar pattern of distribution.

**Figure 6:**
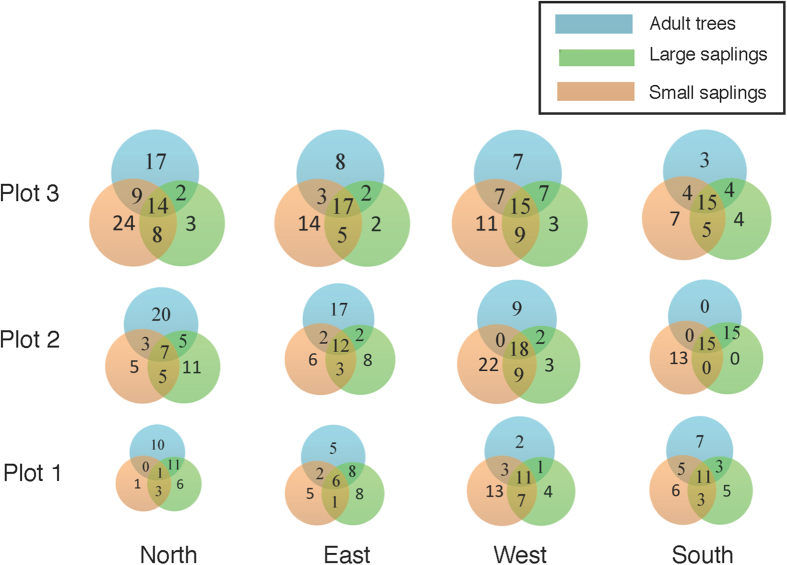
Euler diagram for the number of species in tree size categories (adult tree, large sapling, small sapling) with their overlaps. The area of each circle is dependent on the number of species. North, East, West, and South are the directions from the village boundary lines into the forest. Plot 1 is the nearest site to the villages, Plot 3 is the farthest site from the villages, and Plot 2 is an intermediate distance.

To evaluate the contribution of tree species to the forest structure and biomass of the MDF, we calculated IV (importance value) for each species^5^,^33^,^36^. The IV (importance value) for a species is the sum of the relative frequency (*F*; number of occurrences of the species as a percentage of the total number of occurrences of all species in each L-quadrat), relative density (*D*; number of individuals of a species as a percentage of the total number of individuals of all species in each L-quadrat) and relative dominance (*BA*; total basal area of all trees of one species as a percentage of the total basal area of all species in a plot)^17^ as follows:


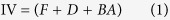


where the maximum values of *F*, *D* and *BA* are 100, respectively. The values of IV were calculated for all woody plants and two functional types (medicinal and pioneer trees) in each plot (see Fig. 3 and Table S2).

The spatial pattern of individuals of the 23 predominant tree species is described using Morisita’s *I*_*δ*_ index in dispersion^39^ as follows:


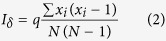


where *q* is the total number of plots (L-quadrat), *N* is the total number of individuals in each species in all L-quadrats, and *x* is the number of individuals of one species in a single plot. Morisita’s *I*_*δ*_ index is independent of sample size and diversity^40^.

The community coefficient for the Bray–Curtis Index (BCI) was used to examine the similarity between plots^41^. Similarity (*S*_*ik*_) between plots *j* and *k* can be expressed as follows:


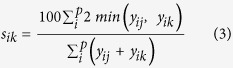


where *y*_*ij*_ and *y*_*ik*_ represent measures of species *i* in the sample plot *j* and *k*, and min (*y*_*ij*_, *y*_*ik*_) is the minimum of *y*_*ij*_ and *y*_*ik*_ and *p* is the number of species. In *S*_*ik*_, Bray and Curtis further simplified this equation based on the species composition and calculated the percentage of similarity, %*S* (A, B), between plots A and B^42^ as follows:


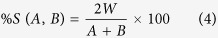


where *W* is the sum of the minimum of IV between plot A and B for each species. The value of A+B represents the sum of *D*, *BA* and *F*; the value is 600 in this study. To compare the percentages of similarity in the forest community between plots, a matrix representing the %*S* and the percent dissimilarity (%DS = 100 − %*S*) between the paired plots is shown in Table S6.

To examine the species diversity of woody plants in each plot, we used the Shannon–Wiener Index (*H*′ = Σ *P*_*i*_ × ln *P*_*i*_)^41^, where *P*_*i*_ is the value of *n*_*i*_ (number of individuals in species *i*)/N (total number of individuals in all species). *H*′ is a measure of overall biodiversity and maximized when all species have the same number of individuals. This index assumes that individuals are randomly sampled from an infinite population. Simpson’s index^43^ is computed as *λ* = Σ*P*_*i*_^2^. Fisher’s index *α* is defined by *S* = *α* ln(1 + *N*/*α*)^44^, where N is the total number of individuals in all species and *S* is the number of species in each L-quadrat. These parameters are measures used to clarify the diversity of tree species by distance from the boundary lines.

To measure the distribution pattern of all individual trees in each plot, the evenness index of the community (*E*) was calculated following the method of Hill (1973)^45^. The values of *E* represent a coherent system for biodiversity that includes the Shannon-Wiener index and Simpson index as follows:


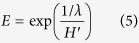


The value of *E* is a useful in determining the evenness of species structures among the plots^46^.

## Additional Information

**How to cite this article**: Popradit, A. *et al.* Anthropogenic effects on a tropical forest according to the distance from human settlements. *Sci. Rep.* **5**, 14689; doi: 10.1038/srep14689 (2015).

## Supplementary Material

Supplementary Information
