# Supplementary material for: Anthropogenic effects on a tropical forest according to the distance from human settlements
Source: Sci Rep. 2015 Oct 5;5:14689. doi: 10.1038/srep14689 (PMC5155698; doi:10.1038/srep14689)
Supplement: Supplementary Information [file srep14689-s1.doc]

**Supplementary information**

**Title:** Anthropogenic effects on a tropical forest according to the distance from human settlements

**Authors:** Ananya Popradit, Thares Srisatit, Somboon Kiratiprayoon, Jin Yoshimura, Atsushi Ishida, Masae Shiyomi, Takehiko Murayama, Pranom Chantaranothai, Somkid Outtaranakorn and Issara Phromma

**Detailed site descriptions and results**

***Specific situation in forest protection in Thailand and South-east Asia***

Currently, the forest damage due to human impacts and global climate change has been obvious in the world including developed and developing countries^1,2^. In Thailand, the annual rate of forest loss in 1995 was ranked in the top ten of tropical countries in the world[^3^](#_ENREF_11). To conserve natural forests, protected areas in Thailand were first established approximately 50 years ago. Thai government has identified a serious deterioration in the ecological service received from natural forests^4,5,6^. Therefore, Thai government has designed a new policy to prevent further deforestation and increase the area of forest cover and it has established network corridors of protected areas in addition to commencing reforestation[^7^](#_ENREF_12). However, human colonization of natural forests from urban areas continues because forests have high economic value in medicinal trees and as a food bank. These new inhabitants have almost no enough knowledge about the nature of these forests and lack a specific skill for sustainable resource use of the forests. Because of this, the degeneration of the important natural tropical forests have been continued in South-east Asia^2^. Therefore, clarifying the human impacts on natural forests is an urgent issue for the protection of the tropical forests in South-east Asia.

***Study site and plot setting***

The current vegetation survey was conducted out in the Phu Koa (PK) area of Phu Kao–Phu Phan Kham National Park (Fig. 1), which is one of o 127 national parks in Thailand. The national park covers 318.4 km^2^ and lies between 16°44′–17°2′N and 102°25′–102°43′E in Nong Bua Lamphu Province, south of Udon Thani Province and north of Khon Kaen Province on the Khorat plateau, Thailand. The PK area is located in the park’s upper northeastern plateau.

The vegetation largely consists of mixed deciduous forests (MDF) along with dry dipterocarp forests and dry evergreen forests. Almost all of the trees of the MDF are dry-deciduous trees that experience an off-leaf period during the dry season^8-12^. Although the area of the park was once 19.7 km^2^ (5.9% in forest area), only 3.07 km^2^ (0.91% in forest area) of intact MDF remained in the national park in 2012^5^. The government of Thailand designated this national park as its 50^th^ national park on September 20, 1985, which was partially to stop the severe degradation of this area caused by an increase in human activity over the preceding 20 years^13^. The Phu Koa Mountain Range consists of two parallel lines of mountains. The outer line is steeper and higher and ranges from 447 m to 580 m a.s.l., whereas the inner line is lower, 313 m to 380 m a.s.l (Fig. S2).

The PK is shaped like a frying pan and has a large plain in its center (Figs. 1 and S2). This area is suspected to overlie an extinct volcano that was active several million years ago. Based on the stratigraphic sequences and fossil correlation, two assemblages of trigoniodid bivalves are indicators of non-marine cretaceous bivalves that once inhabited the area. These two assemblages have been found in the PK and the Khorat Plateau^14^.

The Royal Forestry Department (RFD) and Department of National Park wildlife and Plant Conservation (DNP) in Thailand had attempted to expel village inhabitants from this protected forest. However, several conflicts have occurred, and the RFD and DNP have been unable to expel the inhabitants. After these disputes, the RFD and DNP enacted the boundary lines for protected area within forest. Here, we investigated how the village communities and their activities affect the species composition, species diversity, and natural regeneration of woody plants in the protected area of the MDF.

Three villages (Dong Bak, Wang Mon, and Chai Mongkala) are located in the center of the PK. Using a GPS (Garmin 60Csx) and a topographic map, we established the study plots in all four cardinal directions from the village boundary lines towards the forest (Fig. S2, Table S1). In each direction, we established three plots along a transect line from the village boundary towards the forest. Three plots are located as follows: (1) adjacent to village boundaries, (2) far from village boundaries, and (3) intermediate between the plots. The locations of each plot were slightly adjusted, so that all plots were located only in the MDF. We attempted to space the three plots evenly along each transect (direction), but certain plots were moved intentionally along or away from the transect line until we found an area within the MDF. In total, twelve plots were established (three plots in each direction for the four cardinal directions). The range of elevation among the plots was 235 m to 364 m a.s.l. (Table S1). The straight line from the boundary lines in each plot was derived using a GIS (ArcGIS 10.2.2, Environmental Systems Research Institute).

***Statistical treatments***

To examine the effects of distant on the forest structure, a linear regression analysis was used (Figs. 2, 3, 4 and 5 in the main text), and statistical significance was determined with the Pearson product-moment correlation coefficient (*p* < 0.05). The statistical treatments were conducted with MINITAB (ver. 17, Kozo Keikaku Engineering Inc., Tokyo, Japan) and R (ver. 3.02, R Development Core Team, R Foundation for Statistical Computing, Vienna, Austria; http://www.R-project.org).

**Figure S1** **Importance Value (IV) of the 23 predominant tree species in each plot.**  The IVs in the predominant tree species (IV > 11) in each plot plotted against the distance from the villages boundary to the forest, where IV = D (density) + F (frequency) + BA (basal area). Here, predominant tree species are defined as the primary or secondary dominant species (highest and second highest IV, respectively) in each plot. From left to right and top to bottom, *Cananga odorata* (Annonaceae), *Pterocarpus marcocarpus* (Fabaceae), *Bauhinia saccocalyx* (Fabaceae), *Shorea obtula* (Dipterocarpaceae), *Vitex pinnata* (Verbenaceae), *Xylia xylocarpa* var. *kerrii* (Leguminosae-Mimosoideae), *Shorea siamensis* (Dipterocarpaceae), *Sindora siamensis* (Caesalpiniaceae), *Terminalia corticosa* (Combretaceae), *Hymenodictyon excelsum* (Rubiaceae), *Hymenopyram brachiata* (Lamiaceae), *Anthocephalus chinensis* (Rubiaceae), *Rothmania witti* (Rubiaceae), *Bombax anceps* (Bombacaceae), and *Sterculia guttata* (Sterculiaceae), *Irvingia malayana* (Irvingiaceae), *Pentapterato mentosa* (Combretaceae), *Haldina cordifolia* (Rubiaceae), *Microcos paniculata* (Tiliaceae), and *Dalbergia nigrescens* (papilinoideae), *Casalpiniago defroyana* (Leguminosae), *Dipterocarpus obtusifolius* (Dipterocarpaceae), and *Lagerstoemia floribunda* (Lythraceae).

**Figure S2** **Contour map with the locations of the twelve survey plots**. Twelve survey plots (points with red color) in the study site area. The blue line shows the boundary of three villages constructed within the protected area determined by the RFD and DNP in 2010. Now, the existence of villages is permitted by DNP. However, the expansion of village area and the excess-use of forest resources have been progressed. See Table S5 for topography information of each plot. We make the figure based on a digital map of the Land Development Department in Thailand. Arrow shows the north direction.

**Figure S3 The numbers of pioneer tree species in the twelve survey plots**. There is no significant trend between the numbers and the distance.

**Table S1** **Composition of woody species in the twelve survey plots.** The values in parentheses are for medicinal woody plants. The high values of the variance of frequency indicate that the distribution of all species in the plots is quite different, whereas the low values indicate that all species have a similar pattern of distribution. Significant correlations with the distance (*P* < 0.05 in Pearson’s linear regression) were only found for the number of species and individual density of all individuals. The effects of plant size-class are shown in Figure 3.

| **Plot** | **No. Species (2500 m^−2^)** | **Density (individual ha^−1^)** | **Basal area**  **(m^2^ ha^−1^)** | **Variance of Frequency** |  |
| --- | --- | --- | --- | --- | --- |
| N1 | 32 (12) | 520 | 13.0 | 131 |  |
| N2 | 56 (31) | 1032 | 24.3 | 172 |  |
| N3 | 77 (47) | 2044 | 27.2 | 290 |  |
| E1 | 35 (17) | 964 | 15.6 | 194 |  |
| E2 | 50 (22) | 1208 | 26.7 | 218 |  |
| E3 | 51 (31) | 1644 | 16.1 | 354 |  |
| W1 | 42 (24) | 1304 | 13.9 | 349 |  |
| W2 | 62 (33) | 2180 | 18.2 | 371 |  |
| W3 | 60 (33) | 1740 | 28.2 | 237 |  |
| S1 | 39 (21) | 1180 | 21.3 | 207 |  |
| S2 | 48 (22) | 1264 | 14.7 | 244 |  |
| S3 | 41 (21) | 1896 | 15.6 | 216 |  |
| **Tatal** | **148** | **1416** | **19.6** | **-** |  |

| **code** | **Scientific name** | **N1** | **N2** | **N3** | **E1** | **E2** | **E3** | **W1** | **W2** | **W3** | **S1** | **S2** | **S3** |
| --- | --- | --- | --- | --- | --- | --- | --- | --- | --- | --- | --- | --- | --- |
| **1*** | ***Polyalthia cerasoides (Roxb.) Benth. exBedd.*** | **0** | **1.05** | **6.6** | **0** | **0** | **0** | **0** | **0** | **2.04** | **0** | **0.99** | **1.08** |
| **2*** | ***Erythroxylum cuneatum (Miq.) Kurz*** | **0** | **0** | **0** | **0** | **0** | **0.69** | **0** | **0** | **0** | **0** | **0** | **0** |
| **3*†** | ***Careya sphaericaRoxb.*** | **0** | **1.29** | **1.59** | **0** | **1.02** | **2.55** | **8.91** | **2.13** | **0** | **0** | **3.78** | **0** |
| **4*** | ***Anthocephalus chinensis (Lark )A.Rich.exwalp.*** | **4.71** | **10.89** | **1.5** | **21.39** | **8.07** | **16.2** | **0** | **6.93** | **5.1** | **0** | **0** | **2.34** |
| **5** | ***Dalbergia sp.*** | **0** | **0** | **0** | **0** | **0** | **0** | **0** | **0** | **0** | **2.67** | **0** | **0** |
| **6*•** | ***Irvingia malayanaOliv. ex A. Benn.*** | **0** | **0** | **8.31** | **0** | **0** | **1.38** | **30.33** | **9.09** | **3.3** | **0** | **0** | **0** |
| **7** | ***Haldina cordifolia (Roxb.) Ridsdale*** | **12.03** | **17.46** | **0** | **1.26** | **2.16** | **3.18** | **0.96** | **1.95** | **0** | **3.63** | **4.86** | **1.14** |
| **8*** | ***Lanneaco romandelica*** | **0** | **2.49** | **0.57** | **11.37** | **2.79** | **5.34** | **10.35** | **7.32** | **0** | **4.14** | **0.9** | **6.15** |
| **9** | ***Quercus kingiana Craib*** | **3.15** | **0** | **0.57** | **0** | **0** | **0** | **0.9** | **2.34** | **0** | **0** | **0** | **0** |
| **10** | ***Unknown*** | **0** | **1.08** | **1.17** | **0** | **0** | **0** | **0** | **0** | **0** | **0** | **0** | **0** |
| **11** | ***Unknown*** | **0** | **0** | **2.04** | **0** | **1.08** | **1.38** | **0** | **0** | **0** | **0** | **1.29** | **6.93** |
| **12*** | ***Salacia chinensis L.*** | **0** | **0** | **0** | **0** | **2.01** | **0** | **0.9** | **0.57** | **1.98** | **1.02** | **0** | **0** |
| **13** | ***Harrisonia perforata (Blanco) Merr.*** | **8.82** | **0** | **0** | **0** | **0** | **0** | **1.62** | **0** | **0** | **0** | **0** | **3.93** |
| **14*** | ***Mahonia siamensis Takeda*** | **0** | **0** | **0** | **1.23** | **0** | **0** | **3.06** | **0** | **0** | **3.27** | **1.8** | **1.02** |
| **15*** | ***Streb lusasper Lour.*** | **0** | **0** | **0** | **0** | **0** | **0** | **0** | **0** | **4.89** | **0** | **0** | **0** |
| **16*** | ***Hymenopyramis brachiata Wall. ex Schauer*** | **0** | **1.56** | **6.45** | **3.21** | **17.1** | **2.07** | **0** | **1.17** | **10.29** | **4.47** | **21.3** | **10.41** |
| **17*** | ***Walsura trichostemon Miq.*** | **0** | **0** | **2.49** | **0** | **0** | **0** | **3.18** | **1.5** | **3.51** | **2.28** | **0** | **0** |
| **18*** | ***Antidesma ghaesembilla Gaertn*** | **0** | **1.05** | **3.12** | **0** | **0** | **0.93** | **0.9** | **0.57** | **0** | **0** | **0.99** | **0** |
| **19*** | ***Zollingeria dongnaiensis Pierre*** | **0** | **0** | **0.57** | **0** | **0** | **1.38** | **0** | **0.57** | **0** | **0** | **0** | **0** |
| **20*** | ***Senna siamea (Lam.) Irwin &Barneby*** | **0** | **1.02** | **0** | **0** | **0** | **0** | **0** | **0** | **0** | **0** | **0** | **0** |
| **21*** | ***Cassia garrettiana (Craib.) Inwin&Basneby*** | **2.55** | **2.07** | **0** | **2.88** | **1.23** | **2.37** | **0** | **0** | **1.17** | **1.02** | **0** | **0** |
| **22*** | ***Pavettato mentosa Roxb. ex Smith.*** | **0** | **0** | **0** | **0** | **0** | **0** | **0** | **0** | **0.66** | **0** | **0** | **0** |
| **23** | ***Unknown*** | **2.49** | **2.34** | **0** | **0** | **4.71** | **0** | **0** | **0** | **1.32** | **0** | **5.28** | **4.26** |
| **24*** | ***Smilax spp.*** | **0** | **0** | **0.57** | **0** | **0** | **0** | **0** | **0** | **0** | **0** | **0** | **0** |
| **25*** | ***Microco spaniculata L.*** | **0** | **0** | **21.03** | **0** | **5.34** | **0** | **4.41** | **0.57** | **5.25** | **3.48** | **1.77** | **2.85** |
| **26** | ***Nephelium hypoleucum Kurz*** | **0** | **0** | **0.57** | **0** | **0.99** | **0.69** | **0** | **0** | **0.9** | **0** | **0.9** | **0** |
| **27*** | ***Cassia fistula L.*** | **0** | **0** | **0.57** | **0** | **0** | **0** | **0.9** | **0** | **0** | **0** | **0** | **0** |
| **28** | ***Anomianthus dulcis (Dunal) J.Sinclair*** | **0** | **0** | **0** | **0** | **0** | **0** | **0** | **0** | **4.2** | **0** | **0** | **0** |
| **29*** | ***Smilax bracteataC.Presl subsp. verruculosa*** | **0** | **0** | **0.72** | **0** | **0** | **0** | **0** | **0** | **0.69** | **0** | **0** | **0** |
| **30** | ***Stereospermum fimbriatum (Wall. ex G.Don)*** | **3.99** | **2.43** | **7.53** | **2.7** | **0.99** | **0** | **0** | **0** | **0** | **0** | **4.2** | **1.95** |
| **31*** | ***Bombax anceps Pierre.*** | **0** | **1.2** | **8.43** | **1.23** | **8.31** | **0** | **0** | **0** | **1.32** | **24.36** | **5.16** | **16.95** |
| **32*** | ***Canthiumberberi difolium Geddes*** | **0** | **0** | **1.2** | **0** | **0** | **0** | **0** | **1.74** | **0** | **0** | **0** | **0** |
| **33*** | ***Millettiabran disiana Kurz*** | **0** | **0** | **0.69** | **0** | **0** | **0** | **0** | **0** | **0** | **0** | **4.95** | **4.92** |
| **34*** | ***Diospyros decandra Lour.*** | **0** | **0** | **0** | **0** | **0** | **0** | **0** | **0** | **3.69** | **1.11** | **0** | **0** |
| **35*** | ***Butea monosperma*** | **0** | **0** | **0** | **0** | **0** | **0** | **0** | **0** | **0** | **2.52** | **0** | **0** |
| **36** | ***Dalbergia nigrescensKurz.*** | **25.14** | **0** | **0** | **0** | **0** | **0** | **0** | **0** | **15.57** | **0** | **2.01** | **0** |
| **37*** | ***Ochnaintegerrima (Lour.) Merr.*** | **0** | **0** | **0** | **2.58** | **0** | **6.45** | **2.97** | **2.55** | **1.32** | **0** | **0** | **0** |
| **38*** | ***Diospyros castanea Fletch.*** | **0** | **0** | **0.57** | **0** | **1.05** | **0** | **0** | **1.14** | **1.71** | **0** | **0** | **0** |
| **39** | ***Dalbergia oliveri Gamble ex Prain.*** | **0** | **0** | **1.47** | **0** | **1.05** | **3.24** | **2.76** | **1.47** | **3.96** | **6.33** | **4.53** | **0.81** |
| **40*** | ***Gmelina arboreaRoxb.*** | **0** | **1.08** | **0** | **0** | **0** | **0** | **0** | **0** | **0** | **0** | **0** | **0** |
| **41*** | ***Suregada multiflora (A.Juss.) Baill.*** | **0** | **0** | **5.31** | **0** | **0** | **0** | **0** | **0.66** | **8.64** | **1.11** | **0** | **1.89** |
| **42†** | ***Xylia xylocarpa (Roxb.) Jaub. Var.*** | **16.86** | **19.71** | **5.01** | **11.85** | **8.1** | **24.81** | **4.35** | **12.87** | **2.19** | **5.22** | **8.7** | **1.71** |
| **43** | ***Unknown*** | **0** | **0** | **0.57** | **0** | **0** | **0** | **0** | **0** | **0** | **0** | **0.93** | **0** |
| **44** | ***Anneslea fragrans Wall.*** | **0** | **0** | **0** | **0** | **0** | **0** | **0** | **0** | **0** | **0** | **0.96** | **0** |
| **45*** | ***Schleicheraolosa*** | **1.95** | **4.47** | **0.57** | **0** | **3.39** | **0.69** | **0.93** | **4.56** | **2.82** | **0** | **3.3** | **4.53** |
| **46*** | ***Hopea odorata Roxb.*** | **0** | **0** | **0.57** | **0** | **0** | **0** | **0** | **0** | **0** | **0** | **0** | **0** |
| **47†** | ***Cananga odorata*** | **0** | **34.8** | **41.82** | **48** | **55.68** | **0** | **60.66** | **8.46** | **38.46** | **81.75** | **48.15** | **86.73** |
| **48** | ***Lagerstraemia calyculata. Kurz*** | **10.32** | **0** | **0** | **0** | **0** | **0** | **0** | **0** | **0** | **0** | **0** | **0** |
| **49•** | ***Terminalia corticosa Pierre ex Laness.*** | **6.15** | **0** | **9.93** | **4.71** | **5.28** | **4.98** | **8.22** | **11.94** | **16.47** | **4.53** | **2.94** | **11.64** |
| **50** | ***Lagerstroemia floribunda Jac*** | **0** | **0** | **0** | **0** | **3.15** | **0** | **0** | **0** | **18.78** | **2.19** | **8.52** | **4.83** |
| **51*** | ***Excoecaria oppositifoliaGriff.*** | **0** | **0** | **1.53** | **0** | **0** | **0** | **0** | **0** | **0** | **0** | **0** | **0** |
| **52*** | ***Ehretia laevis Roxb.*** | **0** | **0** | **0** | **0** | **0** | **0** | **0.93** | **0** | **0** | **0** | **0** | **0** |
| **53*** | ***Ellipanthusto mentosus* Kurz var. tomentosus** | **0** | **0** | **1.47** | **0** | **0** | **0** | **3.99** | **9** | **0** | **0** | **0** | **0** |
| **54*** | ***Cratoxylum formosum (Jack) Dyer subsp.*** | **3.9** | **0** | **1.68** | **0** | **0** | **9** | **4.59** | **4.41** | **0** | **0** | **5.22** | **1.56** |
| **55** | ***Cratoxylum cochinchinense (Lour.) Blume*** | **0** | **2.55** | **1.68** | **0** | **0** | **0** | **0** | **0.57** | **0** | **0** | **0** | **0** |
| **56*** | ***Calycopteris floribunda Lamk.)*** | **2.46** | **0** | **0** | **0** | **0** | **0** | **0** | **0** | **0** | **0** | **0** | **0** |
| **57** | ***Vitex pinnata*** | **5.88** | **6.42** | **5.79** | **7.71** | **16.62** | **9.21** | **10.2** | **15.6** | **7.71** | **14.28** | **17.13** | **11.46** |
| **58** | ***Strychnosnux-blanda A.W. Hill*** | **0** | **0** | **0** | **0** | **0** | **0.75** | **0** | **0** | **0** | **0** | **0** | **0** |
| **59†** | ***Shorea obtula*** | **4.2** | **5.76** | **0** | **23.94** | **2.55** | **63.27** | **23.07** | **66.96** | **0** | **0** | **0** | **0** |
| **60*** | ***Miliusave lutina (Dunal) Hook.f. & Thomson*** | **0** | **1.08** | **0** | **0** | **0** | **0** | **0** | **0** | **0** | **0** | **2.34** | **0** |
| **61** | ***Erythrina variegata Linn.*** | **0** | **0** | **0** | **0** | **0** | **0** | **0** | **0** | **0** | **0** | **0** | **9.09** |
| **62*** | ***Xantolis cambodiana (Pierre ex Dubarb) P.Royen*** | **0** | **0** | **0.57** | **0** | **0** | **0** | **0** | **0** | **1.02** | **0** | **1.23** | **0** |
| **63*** | ***Sterculia monosperma Vent*** | **0** | **0** | **0** | **0** | **0** | **0** | **0** | **1.77** | **0** | **0** | **0** | **0** |
| **64*** | ***Bauhinia scandens L. var. horsfieldii (Miq.)*** | **0** | **0** | **0** | **0** | **1.17** | **0** | **0** | **0** | **0** | **0** | **0** | **0** |
| **65** | ***Unknown*** | **0** | **0** | **1.92** | **0.78** | **0** | **4.26** | **1.02** | **0** | **0.66** | **1.14** | **0** | **0** |
| **66†•** | ***Pterocarpus marcocarpus*** | **40.26** | **17.61** | **9** | **52.8** | **33.48** | **29.1** | **56.34** | **26.61** | **8.79** | **21.06** | **26.94** | **19.05** |
| **67*** | ***Grewiae riocarpaJuss.*** | **0** | **1.05** | **0.57** | **0** | **0** | **1.38** | **0** | **3.36** | **0.66** | **0** | **4.41** | **0.78** |
| **68** | ***Sterculia guttataRoxb.*** | **0** | **0** | **7.71** | **5.79** | **0.99** | **0** | **0** | **0.57** | **0.75** | **19.71** | **12.48** | **10.86** |

**Table S2** **Importance Values (IV) from woody plant species in each plot.** The list of all woody plants observed in the study sites (MDF forest) and IVs of each tree species in each plot; the sum of the IV in each tree species is 300 (* indicates medicinal woody plant species, † indicates pioneer woody plant species, and • indicates emergent trees (the max. tree heights > 30m high)).

**Table S2** (Continue)

| **code** | **Scientific name** | **N1** | **N2** | **N3** | **E1** | **E2** | **E3** | **W1** | **W2** | **W3** | **S1** | **S2** | **S3** |
| --- | --- | --- | --- | --- | --- | --- | --- | --- | --- | --- | --- | --- | --- |
| **69** | ***Broussonetia papyrifera*** | **0** | **0** | **0.57** | **0** | **0** | **0** | **0** | **0** | **0** | **0** | **0** | **0** |
| **70** | ***Unknown*** | **4.47** | **1.08** | **1.77** | **0** | **0** | **0** | **0** | **0** | **0.78** | **5.67** | **3.06** | **6.87** |
| **71** | ***Unknown*** | **0** | **0** | **0** | **1.44** | **1.23** | **0** | **0** | **0** | **0** | **0** | **0** | **0** |
| **72*** | ***Croton subiyratusKurz*** | **0** | **0** | **4.53** | **0** | **0** | **0** | **0** | **0** | **0** | **0** | **0** | **0** |
| **73*** | ***Croton oblongifoliusRoxb.*** | **0** | **0** | **0.6** | **0** | **0** | **0** | **0** | **0** | **0** | **0** | **0** | **1.05** |
| **74*** | ***Sauropus androgynus (L.) Merrill.*** | **0** | **1.38** | **0** | **0** | **0** | **0** | **0** | **0** | **0** | **0** | **0** | **0** |
| **75*** | ***Melientha suavis Pierre*** | **0** | **1.02** | **0** | **1.2** | **0** | **1.77** | **0** | **0** | **0** | **0** | **0** | **0** |
| **76*** | ***Thyrsostachys siamensis Gamble*** | **0** | **3.21** | **0** | **0** | **0** | **0** | **0** | **0** | **0** | **0** | **0** | **0** |
| **77*** | ***Caesalpinia pulcherrima (L.) Sw.*** | **0** | **0** | **0.6** | **0** | **0** | **0** | **0** | **0** | **2.25** | **0** | **0** | **1.56** |
| **78*** | ***Albizia lebbeckBenth.*** | **0** | **1.23** | **0** | **2.37** | **2.4** | **0.69** | **0** | **0.78** | **5.37** | **3.75** | **0** | **0** |
| **79†** | ***Dipterocarpus tuberculatusRoxb.*** | **0** | **0** | **4.14** | **0** | **3.15** | **0** | **0** | **1.98** | **0** | **0** | **0** | **0** |
| **80*** | ***Dalbergia cochinchinensis Pierre*** | **0** | **0** | **3** | **0** | **0** | **0** | **1.89** | **0.57** | **0** | **0** | **0** | **0** |
| **81** | ***Erythophleum succirubrum Gagnep.*** | **0** | **0** | **0** | **0** | **1.05** | **0** | **0** | **0** | **0** | **0** | **0** | **0** |
| **82*** | ***Mimusop selengi L.*** | **0** | **0** | **0.57** | **0** | **0** | **0** | **0** | **0** | **0** | **0** | **0** | **0** |
| **83*** | ***Schrebera Swieteniodes Roxb.*** | **0** | **1.05** | **0** | **0** | **0** | **0** | **0** | **0** | **0** | **0** | **0** | **0** |
| **84*†** | ***Spondias bipinnata*** | **0** | **0** | **0** | **0** | **1.17** | **0** | **0** | **0** | **0** | **2.04** | **3.48** | **0** |
| **85** | ***Canarium sabulatum Guillaumin*** | **0** | **0** | **0** | **0** | **0** | **1.47** | **0.9** | **0** | **0** | **0** | **0** | **0** |
| **86*** | ***Phyllanthus emblica Linn.*** | **0** | **0** | **1.77** | **0** | **0** | **2.58** | **0** | **4.14** | **0** | **0** | **0** | **0** |
| **87*** | ***Purging croton, Croton tiglium Linn.*** | **3.06** | **1.44** | **3.54** | **0** | **0** | **0** | **0.9** | **0.57** | **3.36** | **0** | **0** | **0** |
| **88*** | ***Gardenia erythrocladaKurz.*** | **0** | **1.32** | **0** | **0** | **0** | **5.01** | **0** | **0** | **0.75** | **2.85** | **0** | **0** |
| **89•** | ***Sindora siamensisTeijsm.exMiq.*** | **0** | **2.16** | **15.72** | **5.49** | **11.49** | **1.83** | **2.85** | **15.09** | **6.33** | **14.1** | **7.62** | **6.12** |
| **90** | ***Afzelia xylocarpa (Kurz) Craib*** | **0** | **6.84** | **0** | **1.38** | **6.81** | **0** | **0** | **0** | **3.63** | **3.24** | **0.9** | **1.74** |
| **91** | ***Calophyl luminophyllum L.*** | **0** | **0** | **0** | **0** | **0** | **0** | **0** | **0** | **2.85** | **0** | **0** | **0** |
| **92** | ***Mangifera caloneura Kurz*** | **0** | **0** | **0** | **2.79** | **3.12** | **0** | **0** | **0** | **0** | **0** | **0** | **0** |
| **93*** | ***Buchanania latifoliaRoxb.*** | **0** | **0** | **0** | **0** | **0** | **9.03** | **2.01** | **2.31** | **0** | **0** | **0** | **0** |
| **94*** | ***Artocarpus lakoochaRoxb.*** | **0** | **0** | **0.99** | **0** | **0** | **0** | **0** | **0** | **0** | **0** | **0** | **0** |
| **95** | ***Docynia indica (Andr.) Decne.*** | **0** | **2.73** | **0** | **0** | **0** | **1.14** | **0** | **0** | **13.23** | **1.02** | **0.93** | **0** |
| **96** | ***Fagraea fragrans*** | **5.49** | **1.02** | **0** | **0** | **0** | **1.74** | **0** | **0** | **0.84** | **0** | **0** | **0** |
| **97*** | ***Tamilnadia uliginosa (Retz.) Tirveng. &Sastre*** | **0** | **1.5** | **0** | **0** | **0** | **3.27** | **0** | **0** | **0** | **0** | **0** | **0** |
| **98** | ***Morinda coreia*** | **4.02** | **3.75** | **12.3** | **10.62** | **2.1** | **11.19** | **4.59** | **4.38** | **2.22** | **0** | **2.46** | **0** |
| **99** | ***Persea kurziiKosterm.*** | **0** | **0** | **3.21** | **0** | **0** | **0** | **0** | **0** | **1.56** | **0** | **0** | **0** |
| **100*** | ***Pentapterato mentosa Roxb.*** | **6.36** | **11.16** | **0** | **13.65** | **0** | **14.1** | **0.93** | **1.17** | **0** | **1.59** | **0** | **0** |
| **101*** | ***Gluta usitata (Will.) Ding Hou*** | **0** | **1.08** | **0.9** | **2.64** | **0** | **0** | **0** | **4.41** | **0** | **0** | **0** | **0** |
| **102†** | ***Shorea siamensis*** | **46.71** | **23.16** | **0** | **19.95** | **15.09** | **11.97** | **0** | **2.82** | **0** | **0** | **1.05** | **0** |
| **103*** | ***Heterophragma sulfureum Kurz*** | **0** | **0** | **0** | **2.52** | **1.74** | **1.08** | **0** | **0** | **0** | **0** | **2.22** | **0** |
| **104*** | ***Bridelia retusa (L.) A. Juss.*** | **0** | **4.02** | **0** | **1.77** | **0** | **2.58** | **0** | **0.57** | **0** | **1.59** | **0** | **1.68** |
| **105*** | ***Ziziphus oenoplia (L.) Mill. var. oenoplia*** | **0** | **3.99** | **1.77** | **0** | **0** | **0** | **0** | **0.78** | **0.69** | **0** | **0.9** | **2.43** |
| **106** | ***Melia azedarach L.*** | **0** | **1.11** | **1.5** | **1.23** | **0.99** | **5.1** | **2.67** | **0** | **0** | **1.68** | **0** | **0.78** |
| **107*** | ***Hymenodictyon exelsum Wall.*** | **20.52** | **4.62** | **4.83** | **2.97** | **2.34** | **0.69** | **0.9** | **0.78** | **3.81** | **15.18** | **19.65** | **9.33** |
| **108*** | ***Terminalia sp.*** | **1.92** | **0** | **0.6** | **0** | **0** | **0** | **0** | **0** | **0** | **0** | **0** | **0** |
| **109*** | ***Diospyros castanea Fletchev*** | **0** | **7.38** | **0.9** | **2.64** | **2.22** | **0** | **0** | **0** | **1.17** | **1.02** | **0** | **0** |
| **110*** | ***Vitex quinata(Lour.)F.N.Williams*** | **0** | **2.73** | **2.88** | **0** | **0** | **0** | **0** | **0** | **1.14** | **0** | **2.37** | **11.64** |
| **111** | ***Azadirachta excelsa (Jack) Jacobs*** | **0** | **0** | **0** | **0** | **0** | **0** | **0** | **1.71** | **0** | **0** | **0** | **0** |
| **112** | ***Crudia chrysantha, K. Schum*** | **2.01** | **0** | **0** | **0** | **0** | **0** | **0** | **0** | **0** | **0** | **0** | **0** |
| **113** | ***Milletia sp.2*** | **1.92** | **11.1** | **0** | **0** | **10.02** | **0** | **0** | **12.09** | **7.68** | **10.68** | **3.54** | **0** |
| **114** | ***Tetrameles nudiflora R. Br.*** | **0** | **0** | **0** | **0** | **0** | **0** | **0** | **0** | **0** | **0** | **2.19** | **0** |
| **115*** | ***Terminalia bellirica  (Gaertn.) Roxb.*** | **0** | **0** | **0** | **0** | **0** | **0** | **0** | **0** | **0** | **0** | **0** | **0.78** |
| **116** | ***Albizia chinensis (Osbeck) Merr.*** | **2.1** | **1.14** | **0** | **0** | **3.69** | **0** | **0** | **2.13** | **6.96** | **0** | **0.9** | **0.87** |
| **117** | ***Desmos cochinchinensis Lour.*** | **0** | **0** | **1.23** | **0** | **0** | **0** | **0.9** | **1.14** | **0.66** | **0** | **0** | **0** |
| **118*** | ***Crypteronia paniculata Blume.*** | **1.98** | **0** | **0** | **0** | **0** | **0** | **0.9** | **0** | **0** | **0** | **0** | **0** |
| **119*** | ***Pentace burmanicaKurz.*** | **0** | **0** | **1.38** | **0** | **0** | **0** | **0** | **0** | **0** | **0** | **0** | **0** |
| **120** | ***Bauhinia saccocalyx Pierre.*** | **37.92** | **37.02** | **0** | **19.5** | **24.36** | **10.32** | **1.92** | **5.94** | **26.7** | **5.64** | **26.91** | **13.38** |
| **121*** | ***Capparisse piaria Linn.*** | **0** | **0** | **0** | **0** | **0** | **0.69** | **0** | **0** | **0** | **0** | **0** | **0** |
| **122*** | ***Catunaregam tomentosa (Blume ex DC.)Triveng*** | **4.23** | **3.12** | **0** | **3.09** | **2.07** | **3.51** | **1.98** | **0.57** | **0** | **0** | **2.55** | **0** |
| **123*** | ***Caesalpinia godefroyanaO.Kze. C.*** | **0** | **0** | **4.23** | **0** | **1.23** | **0** | **0** | **0** | **0.69** | **16.23** | **6.33** | **11.88** |
| **124** | ***Dialium cochinchinense Pierre*** | **0** | **0** | **1.2** | **0** | **0** | **0** | **0** | **0** | **0** | **0** | **0** | **0** |
| **125** | ***Flacourtiaindica (Burm.f.) Merr.*** | **0** | **0** | **0.99** | **0** | **0** | **0** | **0** | **0** | **0.66** | **0** | **0** | **0** |
| **126*** | ***Terminalia Corticosa Pierre ex laness*** | **0** | **5.43** | **0.57** | **0** | **0** | **3.72** | **0** | **0.57** | **0** | **0** | **0** | **0** |
| **127*** | ***Antidesma ghaesembillaGaertn.*** | **0** | **0** | **0** | **0** | **0** | **0** | **0** | **0.57** | **0** | **0** | **0** | **0** |
| **128*** | ***Rothmania wittii Bremek*** | **0** | **0** | **31.71** | **1.32** | **10.92** | **3.96** | **3.57** | **4.02** | **11.04** | **1.14** | **0** | **0** |
| **129*** | ***Adenanthera pavonina L.*** | **0** | **4.53** | **0.6** | **0** | **0** | **0** | **0** | **0** | **4.08** | **0** | **0** | **0** |
| **130*** | ***Lepisanthes rubiginosa (poxb) Leenh.*** | **0** | **0** | **0** | **0** | **0** | **0** | **0** | **0** | **5.61** | **1.32** | **0** | **0** |
| **131** | ***Eugenia cuminiDruce*** | **0** | **0** | **0.9** | **0** | **0** | **0** | **0** | **0** | **0** | **0** | **0** | **0** |
| **132** | ***Ternstroemia gymnanthera (W. & A.) Bedd.*** | **0** | **1.08** | **0** | **0** | **0** | **0** | **0** | **0** | **0** | **0** | **0** | **0** |
| **133*** | ***Terminalia catappa L.*** | **0** | **0** | **0** | **0** | **0.99** | **0** | **0** | **0** | **0** | **0** | **0** | **0** |
| **134*** | ***Pterolobium integrumCraib*** | **2.28** | **0** | **0.99** | **0** | **0** | **2.34** | **6.63** | **2.37** | **4.14** | **0** | **0** | **0** |
| **135*** | ***Memecylon myrsinoidesBlume*** | **0** | **0** | **6.9** | **0** | **0** | **0** | **0** | **0** | **0** | **0** | **4.92** | **1.02** |
| **136*** | ***Memecylon edule Roxb.*** | **0** | **0** | **0** | **0** | **1.38** | **0** | **1.17** | **0** | **0** | **0** | **0** | **0** |

Table S2 (Continue)

| **code** | **Scientific name** | **N1** | **N2** | **N3** | **E1** | **E2** | **E3** | **W1** | **W2** | **W3** | **S1** | **S2** | **S3** |
| --- | --- | --- | --- | --- | --- | --- | --- | --- | --- | --- | --- | --- | --- |
| **137*†** | ***Dipterocarpus obtusifolius Teijsm.exMiq.*** | **0** | **0** | **0** | **0** | **0** | **2.7** | **18.75** | **16.35** | **1.14** | **0** | **0** | **0** |
| **138*** | ***Unknown*** | **0** | **0** | **0** | **0** | **0** | **0** | **0** | **1.02** | **0** | **0** | **0** | **0** |
| **139** | ***Unknown*** | **0** | **0** | **0.99** | **0** | **0** | **0** | **0** | **0.57** | **0** | **0** | **0** | **0** |
| **140** | ***Dalbergia cultrate Grah.ex Berth.*** | **0** | **7.14** | **0.96** | **0** | **1.02** | **0** | **0** | **0** | **0.93** | **0** | **0** | **0** |
| **141*** | ***Sphenodesme involucrata (Presl) Robinason*** | **0** | **0** | **1.65** | **0** | **0** | **0** | **0** | **0** | **0** | **0** | **0** | **0** |
| **142*** | ***Diospyros ehretioides Wall. ex G. Don*** | **0** | **0** | **0** | **0** | **1.23** | **0.69** | **0** | **0.57** | **0** | **0** | **0** | **0** |
| **143** | ***Anthocephalus chinensis (Lamk.) A. Rich. exWalp.*** | **0** | **0** | **0.81** | **0** | **0** | **0** | **0** | **0** | **0** | **0** | **0** | **0** |
| **144** | ***Wrightiadubia Spreng.*** | **0** | **0** | **0.6** | **0** | **0** | **0** | **0** | **0** | **0** | **0** | **0** | **0** |
| **145*** | ***Unknown*** | **0** | **0** | **0.57** | **0** | **0** | **0** | **0** | **0** | **0** | **0** | **0** | **0** |
| **146** | ***Cochlospermum regium(Mart. &Schrank) Pilg.*** | **0** | **0** | **0** | **0** | **0** | **1.53** | **0** | **0.6** | **0** | **0** | **0** | **0** |
| **147*** | ***Celastruspa niculata Wild.*** | **0** | **0** | **3.57** | **0** | **0** | **0** | **0** | **0** | **0** | **0** | **0** | **0** |
| **148** | ***Gmelinaphilippensis Cham.*** | **0** | **0** | **0** | **0** | **0** | **0** | **0** | **0.57** | **0** | **0** | **0** | **0** |

**Table S3 Species list of the predominant woody plants and their importance value (IV) in each plot**. IV = *D* (relative density) + *F* (relative frequency) + *BA* (relative basal area). In total, 23 woody plants from all plots were considers predominant species with high IVs (IV > 11).

| **Scientific name** | **N1** | **N2** | **N3** | **E1** | **E2** | **E3** | **W1** | **W2** | **W3** | **S1** | **S2** | **S3** |
| --- | --- | --- | --- | --- | --- | --- | --- | --- | --- | --- | --- | --- |
| *Cananga odorata* |  | 34.8 | 41.8 | 48.0 | 55.7 |  | 60.7 |  | 38.5 | 81.7 | 48.2 | 86.7 |
| *Pterocarpus marcocarpus* | 40.3 | 17.6 |  | 52.8 | 33.5 | 29.1 | 56.3 | 26.6 |  | 21.1 | 26.9 | 19.1 |
| *Bauhinia saccocalyx* | 37.9 | 37.0 |  | 19.5 | 24.4 |  |  |  | 26.7 |  | 26.9 | 13.4 |
| *Shorea obtula* |  |  |  | 23.9 |  | 63.3 | 23.1 | 67.0 |  |  |  |  |
| *Shorea siamensis* | 46.7 | 23.2 |  | 19.9 | 15.1 |  |  |  |  |  |  |  |
| *Vitex pinnata* |  |  |  |  | 16.6 |  |  | 15.6 |  | 14.3 | 17.1 | 11.5 |
| *Xylia xylocarpa* | 16.9 | 19.7 |  |  |  | 24.8 |  |  |  |  |  |  |
| *Hymenodictyon exelsum* | 20.6 |  |  |  |  |  |  |  |  | 15.2 | 19.7 |  |
| *Sindora siamensis* |  |  | 15.7 |  |  |  |  | 15.1 |  | 14.1 |  |  |
| *Bombax anceps* |  |  |  |  |  |  |  |  |  | 24.4 |  | 17.0 |
| *Dalbergia nigrescens* | 25.2 |  |  |  |  |  |  |  | 15.6 |  |  |  |
| *Hymenopyramisbrachiata* |  |  |  |  | 17.1 |  |  |  |  |  | 21.3 |  |
| *Terminalia corticosa* |  |  |  |  |  |  |  |  | 16.5 |  |  | 11.6 |
| *Anthocephalus chinensis* |  |  |  | 21.4 |  | 16.2 |  |  |  |  |  |  |
| *Dipterocarpus obtusifolius* |  |  |  |  |  |  | 18.8 | 16.3 |  |  |  |  |
| *Sterculiaguttata* |  |  |  |  |  |  |  |  |  | 19.7 | 12.5 |  |
| *Rothmaniawittiibremek* |  |  | 31.7 |  |  |  |  |  |  |  |  |  |
| *Irvingiamalayana* |  |  |  |  |  |  | 30.3 |  |  |  |  |  |
| *Caesalpiniagodefroyana* |  |  |  |  |  |  |  |  |  | 16.2 |  | 11.9 |
| *Microcospaniculata* |  |  | 21.0 |  |  |  |  |  |  |  |  |  |
| *Lagerstroemia floribunda* |  |  |  |  |  |  |  |  | 18.8 |  |  |  |
| *Haldina cordifolia* |  | 17.5 |  |  |  |  |  |  |  |  |  |  |
| *Pentapteratomentosa* |  |  |  |  |  | 14.1 |  |  |  |  |  |  |

**Table S4** The distribution pattern of individuals of 23 predominant woody plants species (IV>11). A value of 1 in Morisita’s index (*I_δ_*) indicates a random distribution, values >1 indicate a clumped distribution, and values <1 indicate a uniform or regular distribution.

| **Scientific name** | **Family** | *I_δ_* |
| --- | --- | --- |
| *Cananga odorata* | Annonaceae | 1.8 |
| *Pterocarpus marcocarpus* | Fabaceae | 1.3 |
| *Shorea obtula* | Dipterocarpaceae | 4.1 |
| *Vitex pinnata* | Verbenaceae | 1.6 |
| *Bauhinia saccocalyx Pierre.* | Fabaceae | 1.5 |
| *Sindora siamensis* | Caesalpiniaceae | 1.7 |
| *Lagerstroemia floribunda* | Lythraceae | 3.8 |
| *Terminalia corticosa* | Combretaceae | 1.8 |
| *Dalbergia nigrescens* | Papilinoideae | 4.0 |
| *Hymenopyramis brachiata* | Lamiaceae | 2.2 |
| *Bombax anceps* | Bombacaceae | 3.1 |
| *Sterculia guttata* | Sterculiaceae | 2.8 |
| *Haldina cordifolia* | Rubiaceae | 1.6 |
| *Anthocephalus chinensis* | Rubiaceae | 2.2 |
| *Dipterocarpus obtusifolius* | Dipterocarpaceae | 6.0 |
| *Irvingia malayana* | Rvingiaceae | 6.0 |
| *Pentaptera tomentosa* | Combretaceae | 3.3 |
| *Caesalpinia godefroyana* | Leguminosae | 2.9 |
| *Hymenodictyon excelsum* | Rubiaceae | 1.7 |
| *Shorea siamensis* | Dipterocarpaceae | 3.0 |
| *Xylia xylocarpa* | Leuminosae-Mimosoideae | 1.9 |
| *Rothmania wittii* | Rubiaceae | 5.3 |
| *Microcos paniculata* | Tiliaceae | 5.5 |

**Table S5 Values of the Shannon–Wiener Index (*H’*), Fisher’s α, 1-Simpson’s *λ,* and Hill’s Evenness (*E*) indexes in each survey plot.** *H’*, α, and (*1-λ*) are indexes of the biodiversity of woody plants, and *E* is an index of the evenness of the distribution pattern of all individual trees. See the main text for the equations.

| **Plot** | **Distance** | ***H’*** | ***α*** | ***1-λ*** | ***E*** |
| --- | --- | --- | --- | --- | --- |
| N1 | 310.00 | 4.15 | 13.56 | 0.92 | 0.20 |
| N2 | 692.00 | 4.73 | 22.02 | 0.94 | 0.14 |
| N3 | 1369.59 | 4.83 | 25.18 | 0.92 | 0.10 |
| E1 | 223.81 | 4.03 | 10.80 | 0.90 | 0.17 |
| E2 | 479.92 | 4.31 | 17.08 | 0.90 | 0.90 |
| E3 | 687.74 | 4.44 | 15.34 | 0.90 | 0.14 |
| W1 | 514.85 | 3.99 | 12.83 | 0.87 | 0.13 |
| W2 | 893.00 | 4.53 | 18.42 | 0.91 | 0.15 |
| W3 | 950.00 | 4.93 | 18.87 | 0.94 | 0.13 |
| S1 | 390.11 | 4.07 | 12.04 | 0.89 | 0.15 |
| S2 | 585.55 | 4.47 | 15.75 | 0.92 | 0.14 |
| S3 | 923.40 | 3.87 | 10.77 | 0.84 | 0.13 |

**Table S6** **Topography of the survey plots.** Abbreviations in plots: N, E, W and S indicate north, east, west, and south from the village boundary to the forest, respectively.

| **Plots in cardinal direction** | | **Topography** | |
| --- | --- | --- | --- |
| *North* | UTM coordinate  zone 48Q | Distance from the village  boundary lines (m) | Elevation (m ASL) |
| N1 | 230111 1876584 | 310 | 256 |
| N2 | 230277 1876780 | 692 | 290 |
| N3 | 230503 1877600 | 1370 | 364 |
| East |  |  |  |
| E1 | 231455 1874233 | 224 | 279 |
| E2 | 231615 1874313 | 480 | 290 |
| E3 | 231190 1874112 | 688 | 268 |
| West |  |  |  |
| W1 | 228450 1875531 | 515 | 272 |
| W2 | 228202 1875830 | 893 | 286 |
| W3 | 228051 1875554 | 950 | 270 |
| South |  |  |  |
| S1 | 229227 1870986 | 390 | 248 |
| S2 | 229119 1870750 | 586 | 235 |
| S3 | 229244 1870324 | 924 | 268 |

**Table S7 Percentage similarity and percentage dissimilarity in species composition among plots.** To examine the similarity or dissimilarity between plots, Bray and Curtis Index (BCI) values were used to determine a standardized species score to combine the measurements of IV (D + BA + F) in woody plant species. The upper right section and the lower left section show the similarity and dissimilarity between two plots, respectively. For the similarity values, the minimum value was 17.1 between Plot N1 and N3, indicating that the species compositions in these plots presented the greatest differences of all plot pairs, and the maximum value was 68.9 between Plot S1 and S3, indicating a similar species composition. The similarity in forest structure between plots appears to be independent of both direction and distance.

|  | **N1** | **N2** | **N3** | **E1** | **E2** | **E3** | **W1** | **W2** | **W3** | **S1** | **S2** | **S3** |
| --- | --- | --- | --- | --- | --- | --- | --- | --- | --- | --- | --- | --- |
| **N1** |  | 51.3 | 17.1 | 44.7 | 39.6 | 38.6 | 27.7 | 30.7 | 30.5 | 23.0 | 40.7 | 25.4 |
| **N2** | 48.7 |  | 31.0 | 57.1 | 54.8 | 43.9 | 30.6 | 35.1 | 43.0 | 36.4 | 46.8 | 36.9 |
| **N3** | 82.9 | 69.1 |  | 35.6 | 43.7 | 21.4 | 38.0 | 32.5 | 44.6 | 41.6 | 44.1 | 43.7 |
| **E1** | 55.3 | 43.0 | 64.5 |  | 61.6 | 55.6 | 57.4 | 43.2 | 37.2 | 42.9 | 48.5 | 43.2 |
| **E2** | 60.4 | 45.2 | 56.3 | 38.4 |  | 37.1 | 46.6 | 43.4 | 54.6 | 56.0 | 64.4 | 54.0 |
| **E3** | 61.4 | 56.1 | 78.6 | 44.4 | 63.0 |  | 39.4 | 60.2 | 22.2 | 24.2 | 29.6 | 22.1 |
| **W1** | 72.3 | 69.4 | 62.0 | 42.6 | 53.6 | 60.6 |  | 51.9 | 33.7 | 43.0 | 40.4 | 40.7 |
| **W2** | 69.3 | 64.9 | 67.5 | 56.8 | 56.6 | 39.9 | 48.1 |  | 31.2 | 33.7 | 35.5 | 29.0 |
| **W3** | 69.5 | 57.1 | 55.4 | 62.8 | 45.4 | 77.8 | 66.3 | 68.8 |  | 40.7 | 48.4 | 43.3 |
| **S1** | 77.0 | 63.6 | 58.4 | 57.1 | 44.0 | 75.8 | 57.1 | 66.3 | 59.4 |  | 57.9 | 68.9 |
| **S2** | 59.3 | 53.2 | 56.0 | 51.5 | 35.6 | 70.4 | 59.6 | 64.6 | 51.7 | 42.1 |  | 61.2 |
| **S3** | 74.6 | 63.1 | 56.4 | 56.8 | 46.0 | 77.9 | 59.3 | 71.0 | 56.7 | 31.1 | 38.8 |  |

**References**

1 Puhe, J. & Ulrich B. *Global Climate Change and Human Impacts on Forest Ecosystems*: *Postglacial Development, Present Situation, and Future Trends in central Europe*. (eds. Puhe, J. & Ulrich B.) Ecological Studies **143**, 892 P (Springer-Verlag Berlin Heidelberg, 2001).

2 Guhardja, E., Fatawi M., Sutisna, M., Mori, T. & Ohta, S. (eds.) *Rainforest Ecosystems of East Kalimantan*: *El Niño, Drought, Fire and Human Impacts*. (eds. Guhardja, E. *et al.*) *Ecological Studies* **140**, 330 P (Springer-Verlag Tokyo, 2000).

3 Bajracharya, S. B., Gurung, G. B. & Basnet, K. Learning from community participation in conservation area management. *J. Forest Livelihood* **6**, 54-66 (2007).

4 Santisuk, T. *et al.* *Plants for our Future: Botanical Research and Conservation Needs in Thailand.* (Royal Forest Department, Bangkon, Thailand, 1991).

5 Myers, N., Mittermeier, R. A., Mittermeier, C. G., Da Fonseca, G. A. & Kent, J. Biodiversity hotspots for conservation priorities. *Nature* **403**, 853-858 (2000).

6 Stibig, H. J. *et al.* A land‐cover map for South and Southeast Asia derived from SPOT‐VEGETATION data. *J. Biogeogr.* **34**, 625-637 (2007).

7 Trisurat, Y. Applying gap analysis and a comparison index to evaluate protected areas in Thailand. *Environ. Manage.* **39**, 235-245 (2007).

8 Bunyavejchewin, S. Analysis of the tropical dry deciduous forest of Thailand: I. Characteristics of the dominance-types. *Nat. Hist. Bull. Siam Soc* **31**, 109-122 (1983).

9 Ashton, P. Toward a regional classification of the humid tropics of Asia. *Tropics* **1**, 1-12 (1991).

10 Blasco, F., Bellan, M. & Aizpuru, M. A vegetation map of tropical continental Asia at scale 1: 5 million. *J. Veg. Sci.* **7**, 623-634 (1996).

11 Marod, D., Kutintara, U., Yarwudhi, C., Tanaka, H. & Nakashisuka, T. Structural dynamics of a natural mixed deciduous forest in western Thailand. *J. Veg. Sci.* **10**, 777-786 (1999).

12 Ishida, A. *et al.* Seasonal variations of gas exchange and water relations in deciduous and evergreen trees in monsoonal dry forests of Thailand. *Tree Physiol.* **30**, 935-945 (2010).

13 Department of National Parks, W. a. P. C. Statistic data. (2012).

14 Sha, J., Pan, Y., Wang, Y., Zhang, X. & Rao, X. Non-marine and marine stratigraphic correlation of Early Cretaceous deposits in NE China, SE Korea and SW Japan, non-marine molluscan biochronology, and palaeogeographic implications. *J. Stratigraphy* **36**, 357-381 (2012).
